# Predicting the Potential Suitable Habitat of *Solanum rostratum* in China Using the Biomod2 Ensemble Modeling Framework

**DOI:** 10.3390/plants14172779

**Published:** 2025-09-05

**Authors:** Jiajie Wang, Jingdong Zhao, Lina Jiang, Xuejiao Han, Yuanjun Zhu

**Affiliations:** 1 Institute of Ecological Conservation and Restoration, Chinese Academy of Forestry, Beijing 100091, China; 2 Forestry and Grassland Work Station of Inner Mongolia, Hohhot 010011, China

**Keywords:** *Solanum rostratum* Dunal, Biomod2, biological invasion, potential distribution, ensemble model

## Abstract

*Solanum rostratum* Dunal is a highly invasive species with strong environmental adaptability and reproductive capacity, posing serious threats to agroforestry ecosystems and human health. In this study, we compiled occurrence records of *S. rostratum* in China from online databases and sources in the literature. We employed the Biomod2 ensemble modeling framework to predict the potential distribution of the species under current climatic conditions and four future climate scenarios (SSP126, SSP245, SSP370, and SSP585), and to identify the key environmental variables influencing its distribution. The ensemble model based on the committee averaging (EMca) approach achieved the highest predictive accuracy, with a true skill statistic (TSS) of 0.932 and an area under the curve (AUC) of 0.990. Under present climatic conditions, *S. rostratum* is predominantly distributed across northern China, particularly in Xinjiang, Inner Mongolia, and the northeastern provinces, covering a total suitable area of 1,191,586.55 km^2^, with highly suitable habitats accounting for 50.37% of this range. Under future climate scenarios, the species’ suitable range is projected to expand significantly, particularly under the high-emissions SSP585 scenario, with the distribution centroid expected to shift significantly toward high-altitude regions in Gansu Province. Precipitation and temperature emerged as the most influential environmental factors affecting habitat suitability. These findings indicate that ongoing global warming may facilitate the survival, reproduction, and rapid spread of *S. rostratum* across China in the coming decades.

## 1. Introduction

Biological invasion refers to the phenomenon in which species, through natural dispersal or human-mediated activities, are introduced into non-native habitats, subsequently establish populations, expand their distribution, and ultimately pose a threat to local ecological balance [1]. Although some invasive plants may contribute to increased biodiversity, landscape enhancement, or economic value [2]—for instance, *Ipomoea purpurea* (L.) Roth and *Oxalis corymbosa* DC. are commonly cultivated as ornamental plants, while *Medicago sativa* L., *Bromus japonicus* Thunb., *Trifolium repens* L., and *Lolium perenne* L. are recognized as high-quality forage species—their negative impacts on ecosystems remain a serious concern. Numerous alien invasive plants have altered the biogeographic distribution of native species as well as the structure and function of natural ecosystems, thereby posing significant threats to ecological security and leading to substantial economic losses [3,4]. In China alone, economic losses caused by invasive alien plants exceed CNY 50 billion annually [5]. Once successfully established, invasive species are exceedingly difficult to eradicate and require long-term management with considerable financial investment. Consequently, the prevention and control of invasive alien plants has become a pressing global ecological challenge of the 21st century, attracting widespread international attention [6].

Species distribution models (SDMs) have become an essential tool in ecological research, primarily used to quantify the relationship between species and environmental variables and to predict potential geographic distribution patterns [7]. In recent years, SDMs have played an increasingly prominent role in the study of invasive plants, offering valuable insights into the ecological mechanisms driving invasions. By integrating species occurrence data with environmental factors such as climate and topography, SDMs can identify key environmental variables that determine species distribution patterns [7,8]. Although numerous SDMs have been developed, each with distinct advantages and focuses, relying on a single model often limits predictive accuracy and generalizability. Ensemble modeling, which combines the outputs of multiple SDMs, has been shown to significantly improve prediction accuracy [9]. Researchers have successfully employed ensemble SDMs for various applications, including modeling the potential spatial distribution of invasive species [10], identifying suitable habitats [11], and assessing invasion risk [12] and disturbance intensity [13]. These studies consistently demonstrate that ensemble approaches can effectively overcome the limitations of individual models, resulting in more robust, accurate, and reliable predictions [14].

**Figure 1:**
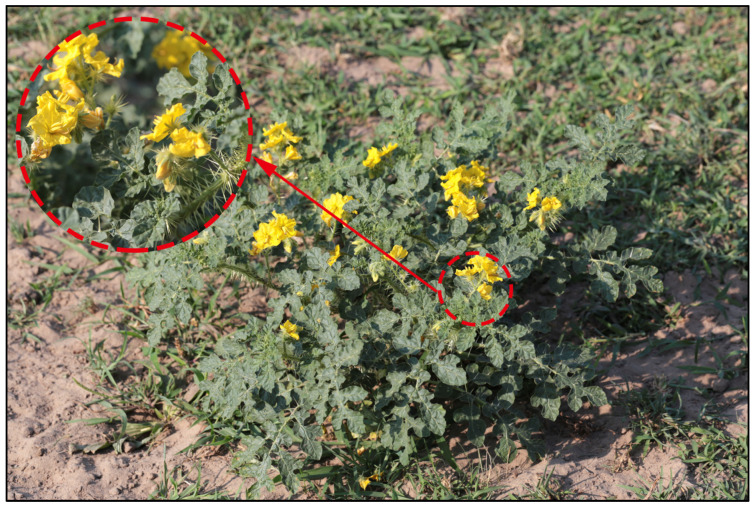
Wild status of *Solanum rostratum* Dunal in China.

*Solanum rostratum* Dunal (Figure 1), a highly aggressive invasive weed native to North America, displays exceptional ecological adaptability along with strong reproductive and dispersal capabilities; it thrives in both arid and humid environments and is widely recognized as a highly aggressive alien species. Previous studies have shown that *S. rostratum* significantly inhibits tomato seed germination and early seedling growth [15]. Moreover, *S. rostratum* serves as a major host for agricultural pests such as the Colorado potato beetle (*Leptinotarsa decemlineata*) and the potato cyst nematode (*Globodera rostochiensis*), providing both feeding and oviposition sites [16], thereby increasing the risk of pest outbreaks and crop damage. Alarmingly, *S. rostratum* produces solanine, a neurotoxic alkaloid, which can cause severe poisoning and even death in livestock that consume it. In addition to threatening animal health, solanine contamination may reduce the quality of agricultural by-products such as wool, posing risks to both the agricultural economy and human health [17]. Beyond its direct impacts on agriculture and health, *S. rostratum* also competes with native species for resources, potentially driving local extinctions and reducing biodiversity. Owing to its severe ecological impacts, it has been labeled an “ecological killer”.

*S. rostratum* was first recorded in China in 1981 in Chaoyang District, Liaoning Province [18]. Since then, it has been reported in multiple other regions, including Baicheng (Jilin) [19] and Zhangjiakou (Hebei) [20]. Between 2005 and 2009, the species was detected in Urumqi, Shihezi, and Changji in the Xinjiang Uygur Autonomous Region, where it has gradually expanded its range. These records indicate that *S. rostratum* has already established a clear distribution pattern across northern China. Previous studies have demonstrated that *S. rostratum* exhibits strong adaptability to arid environments, facilitating its successful colonization and continued spread [18]. Notably, regions with relatively abundant water resources, such as oasis ecosystems, appear particularly vulnerable to invasion by this species, likely due to its ecological preference for moist conditions. This indicates that *S. rostratum* possesses broad ecological amplitude and may have the potential to spread further south in China. Without timely and effective control measures, the species could spread extensively and pose a significant risk of large-scale outbreaks. Although many studies have explored the reproductive biology, seed germination, and ecophysiological traits of *S. rostratum* [21], its potential distribution across China remains poorly understood. This lack of spatial prediction data hinders the development of targeted and efficient management strategies. Therefore, this study aims to model the potential distribution of *S. rostratum* under current and projected climate scenarios, identify the key environmental drivers of its distribution, and provide a scientific foundation for informed prevention and control strategies in China.

Previous research has primarily used SDMs to predict the potential range of *S. rostratum* in specific regions of China [22,23,24]. However, global-scale habitat suitability assessments have been conducted at relatively coarse resolutions [25,26], limiting the evaluation of invasion risk at the provincial level and constraining the development of effective monitoring and management strategies. In recent years, *S. rostratum* has been increasingly reported in Beijing and Hebei, and continues to spread across Xinjiang, Inner Mongolia, and other regions, underscoring a clear and accelerating expansion trend in China. Accordingly, there is an urgent need to conduct a nationwide assessment of its suitable habitat range to support targeted prevention and control efforts. In this study, we employed an ensemble species distribution modeling approach that integrates multiple algorithms to enhance prediction accuracy. For the first time, we modeled the potential distribution of *S. rostratum* across China under both current and future climate scenarios (2030s, 2050s, 2070s, and 2090s). Specifically, this study aimed to evaluate the current climatic suitability and identify the key environmental variables influencing the distribution of *S. rostratum*, forecast changes in suitable habitats under four projected future climate scenarios, and analyze the spatial distribution patterns and centroid shifts of its potential range over time. These findings are intended to support evidence-based strategies for monitoring and managing the invasion of *S. rostratum* in China.

## 2. Results

### 2.1. Comparison of Model Performance

**Figure 2:**
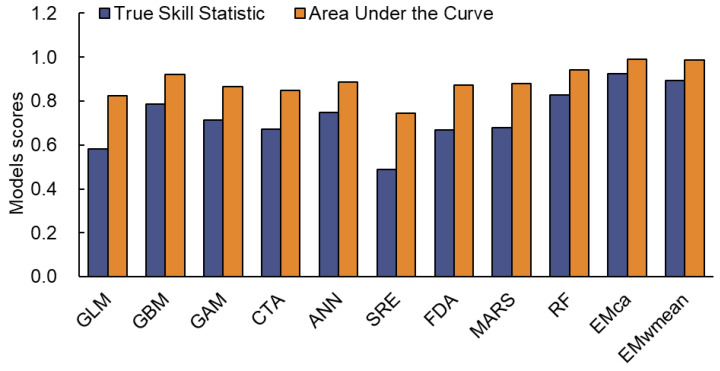
The performance evaluation of individual and ensemble models used for predicting the potential distribution of *S. rostratum*.

The predictive performance of the ten individual models embedded within the Biomod2 package was evaluated using two widely accepted metrics: the true skill statistic (TSS) and the area under the receiver operating characteristic curve (AUC) (Figure 2). Higher TSS values (approaching 1) indicate greater predictive accuracy [27]. AUC scores assess the model’s ability to distinguish between presence and absence: values around 0.5 suggest random prediction, values between 0.8 and 0.9 indicate good performance, and values above 0.9 reflect excellent discriminative power [28]. Among all individual models, the random forest (RF) algorithm exhibited the highest predictive performance, achieving an optimal balance between sensitivity and specificity. To enhance predictive accuracy and minimize model uncertainty, the committee averaging (EMca) ensemble approach was employed to integrate outputs from individual models. The resulting ensemble model demonstrated the highest predictive performance, with a TSS of 0.932 and an AUC of 0.990, significantly surpassing any single algorithm. These results indicate that the ensemble model provides superior reliability in simulating the potential distribution of *S. rostratum*.

### 2.2. Current Potential Distribution of S. rostratum in China

**Figure 3:**
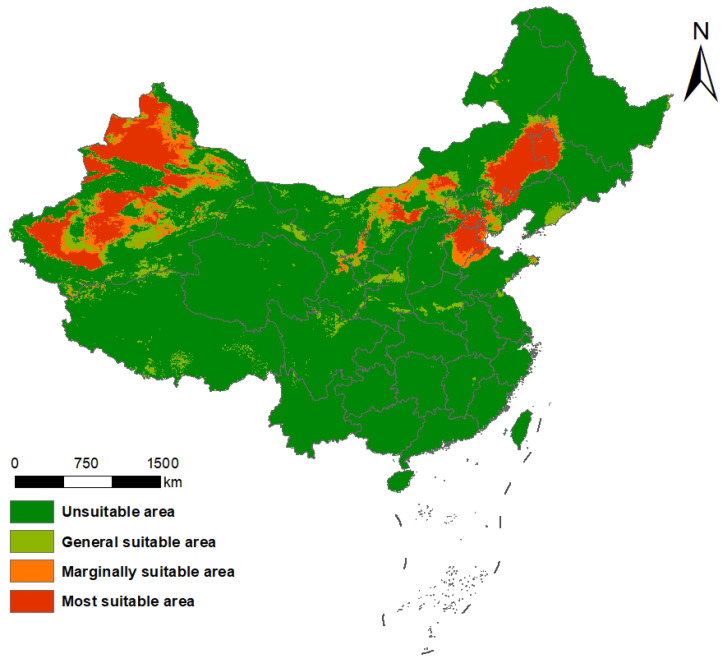
Current potential suitable distribution of *S. rostratum* in China.

Based on the ensemble model projections (Figure 3), the current potential distribution of *S. rostratum* is primarily concentrated in northern China. The most suitable areas for *S. rostratum* in China are concentrated in parts of Xinjiang, central–western Inner Mongolia, and northern North China (northern Hebei and parts of Shanxi), with additional suitable habitats in Heilongjiang, Jilin, Liaoning, Beijing, Tianjin, Shandong, Gansu, and Ningxia. Marginally suitable areas mainly occur as transitional belts surrounding these core zones, including extensions toward northwestern China and peripheral regions of North China, with scattered occurrences in northern Sichuan and parts of Shaanxi. Moderately suitable habitats appear as small, fragmented patches with irregular distributions, occurring sporadically in several regions, particularly in parts of southwestern and southeastern China.

The total suitable habitat area under current climatic conditions is estimated at 1,191,586.55 km^2^, accounting for approximately 12.4% of China’s total land area (Table 1). Of this, highly suitable zones are predominantly located in Xinjiang, Gansu, Ningxia, Inner Mongolia, Jilin, Tianjin, Beijing, and Hebei, totaling 600,215.76 km^2^—about 50.37% of the overall suitable area. These findings suggest a substantial potential for the species to establish and expand in northern and northwestern China under present climate conditions.

### 2.3. Projected Future Distribution of S. rostratum Under Climate Change Scenarios

The results (Table 1) indicate notable spatial and temporal variation in habitat suitability in response to different climate change scenarios. Under the SSP126 scenario (low forcing), the total suitable area remains relatively stable across all four future periods, with only a slight increase of 7449.01 km^2^ (approximately 0.31%) in the 2070s compared to the 2050s, indicating minimal sensitivity to climate change in this scenario. In contrast, under the SSP245 scenario (moderate forcing), the total suitable area experiences a slight decline by the 2090s compared to the 2070s. Notably, the highly suitable habitats shrink by 61,709.11 km^2^, a decrease of 3.17%, suggesting a shift in optimal habitat conditions. The SSP370 scenario (moderate-to-high forcing) shows the most significant increase in suitable habitat by the 2030s, with a total gain of 1,199,947.14 km^2^ relative to the current climate, indicating that warmer conditions may initially favor the expansion of *S. rostratum*. Under the SSP585 scenario (high forcing), the species shows both high sensitivity and volatility. Although the 2030s exhibit the smallest gain in total suitable area compared to the current climate (+741,571.22 km^2^), the overall variation across time is the largest among all scenarios. By the 2050s, the total suitable area increases by 747,501.55 km^2^, a 3.87% rise from the 2030s. However, in the 2070s, suitable habitats experienced a substantial contraction, with a loss of 470,890 km^2^, corresponding to a habitat reduction rate as high as 39.232%. Across all four climate scenarios, projections for the 2030s consistently show more than 100% increases in total suitable area compared to current conditions, particularly under higher emission pathways. This suggests that rising greenhouse gas concentrations may significantly enhance the expansion potential of *S. rostratum*, underscoring the importance of climate change in shaping its future distribution dynamics.

### 2.4. Habitat Centroid Shift and Spatial Pattern Dynamics

**Figure 4:**
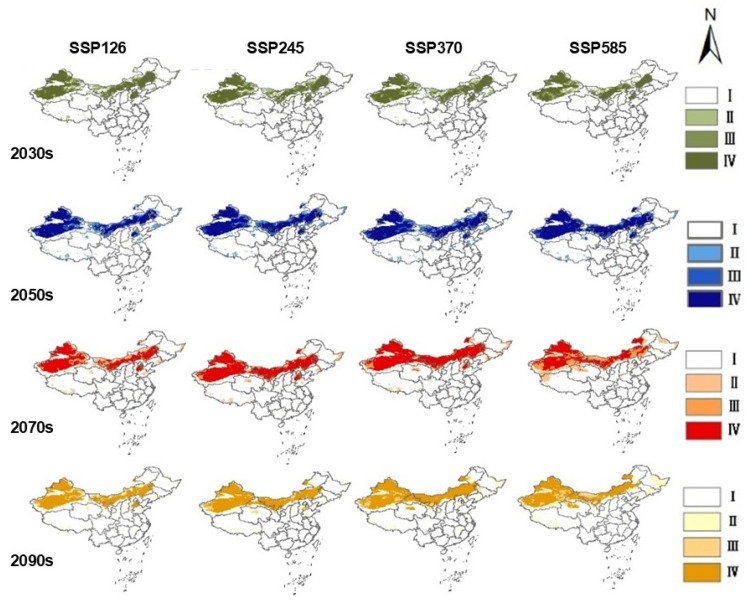
Potential distribution map of *S. rostratum* in China under different climate scenarios based on integrated model prediction. I: Unsuitable area; II: lowly suitable area; III: moderately suitable area; IV: highly suitable area.

According to the ensemble model projections (Figure 4), the total suitable habitat of *S. rostratum* in China is expected to expand under all four future climate scenarios. Notably, the highly suitable areas exhibit the most significant increase, indicating that future climate conditions will likely become more favorable for the survival and spread of this invasive species. As time progresses, the distribution range of *S. rostratum* may shift southward, indicating an increased risk of invasion in southern China. Furthermore, the rate of expansion is positively correlated with greenhouse gas emission levels, highlighting a strong link between climate change and biological invasion risk. This reinforces the urgency of integrating climate projections into invasive species risk assessments and management strategies.

### 2.5. Habitat Centroid Dynamics Under Future Climate Scenarios

**Figure 5:**
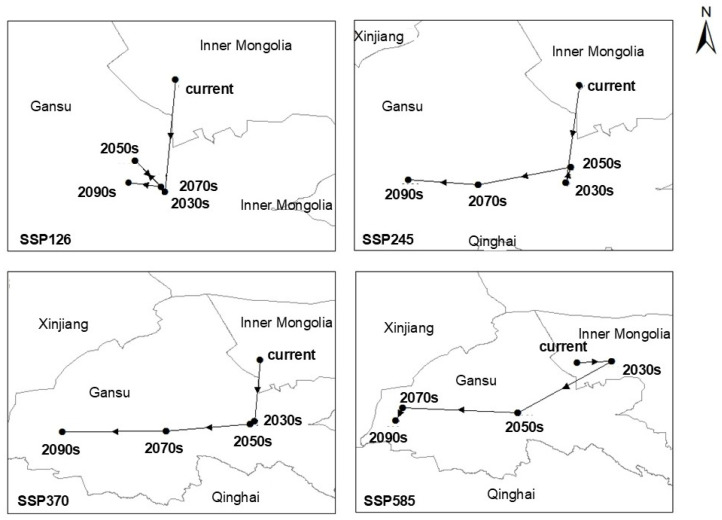
Centroid migration routes under different climate change scenarios.

In terms of spatial pattern dynamics (Figure 5), the centroid of suitable habitat for *S. rostratum* exhibits divergent trajectories under different climate scenarios. Under the SSP126 scenario, the centroid exhibits a minor southward shift and remains relatively stable near its 2030s position. By contrast, under the SSP245, SSP370, and SSP585 scenarios, the centroid steadily shifts southward and westward over time.

At present, the centroid of suitable habitat is located in Inner Mongolia. By the 2030s, it shifts southward to Gansu Province under all scenarios except SSP585. Under the SSP585 scenario, the centroid shifts southwestward and does not reach Gansu until the 2050s. Overall, the centroid exhibits a clear temporal trend of southward and westward movement, with the extent of displacement increasing under higher greenhouse gas emission levels—highlighting the strong influence of climate forcing on the potential invasion range of *S. rostratum*.

### 2.6. Key Environmental Predictors of S. rostratum Distribution

**Figure 6:**
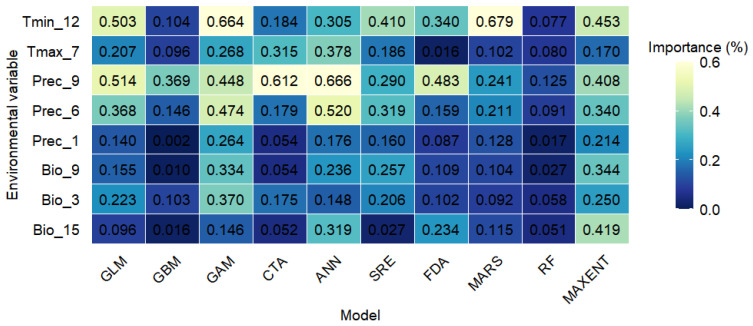
Environmental variable importance for predicting the distribution of *S. rostratum* across individual models in the ensemble models.

Using the Biomod2 package, we selected eight environmental variables to model the potential distribution of *S. rostratum*, including isothermality, mean temperature of the driest quarter, precipitation seasonality, monthly precipitation in January, June, and September, maximum temperature in July, and minimum temperature in December. The results (Figure 6) show that among all bioclimatic and precipitation variables, minimum temperature in December (Tmin_12) and precipitation in September (Prec_9) consistently exhibit high importance across most models—particularly in RF, the boosted regression tree model (GBM), and artificial neural networks model (ANNs)—suggesting that they are key drivers of the potential distribution of *S. rostratum*. In contrast, variables such as Bio_15 and Prec_1 showed relatively low contributions in most models, indicating limited predictive power.

## 3. Discussion

### 3.1. Dispersal Potential of S. rostratum and Future Changes in Suitable Habitats

*S. rostratum* is a highly adaptable invasive species that reproduces solely by seed. Its spiny, small, and lightweight seeds enable long-distance dispersal and remain viable under harsh conditions [29]. Coupled with abundant flowering and attraction of diverse pollinators [30], these traits confer high reproductive success and rapid spread across both arid and humid environments. As shown in Figure 5, the centroid of suitable habitat for *S. rostratum* is projected to shift from Inner Mongolia to Gansu Province’s higher-altitude regions under future climate scenarios. Under the SSP126 and SSP245 pathways, the centroid shifts to central Gansu; under SSP370 and SSP585, it continues westward, reaching the province’s western margins. These findings suggest that the extent of centroid migration is positively correlated with climate forcing intensity, indicating greater habitat redistribution under more extreme climate change. Additionally, all four climate scenarios project a substantial expansion in total suitable area by the 2030s, with increases exceeding 100% compared to current levels. This supports the conclusion that global warming significantly facilitates the spread of *S. rostratum*, increasing its ecological threat. These results are consistent with recent modelling efforts by Huang et al. (2024) [31], who reported southward shifts and habitat expansion under multiple SSP scenarios. Table 1 further shows that habitat gains outpace losses across all future scenarios, with a notable southward expansion trend.

Nevertheless, despite the multiple management strategies that have been applied to control invasive plants, significant challenges persist in practice. Chemical control may cause environmental risks and foster resistance, mechanical removal is difficult to maintain over large areas, and biological control is limited by long establishment periods and potential ecological risks. As a result, once invasive species become widely established, complete eradication is often unfeasible, and ongoing management is required to mitigate their impacts. In this context, predicting the potential suitable habitats of *S. rostratum* can reveal areas most favorable for its establishment, help identify high-risk regions, optimize resource allocation, and provide a scientific basis for early warning and intervention.

### 3.2. Climatic Drivers of Habitat Suitability

Temperature and precipitation are not only key indicators of regional climatic conditions but also directly influence plant growth and development by altering physiological processes and biochemical pathways [32]. In this study, eight environmental variables with low multicollinearity (VIF < 10) were retained, among which Tmin_12 and Prec_9 were identified as the most influential factors shaping the distribution of *S. rostratum*. Previous studies have demonstrated that temperature significantly affects the seed germination of *S. rostratum*, with optimal germination observed within a specific temperature range [33]. This suggests that temperature fluctuations could alter germination success and, consequently, establishment rates. Moreover, *S. rostratum* is known to tolerate both arid and humid environments and is particularly suited to cooler climatic zones, consistent with its current distribution concentrated in northern China. As an annual species that reproduces sexually, the persistence and expansion of *S. rostratum* populations are largely dependent on fruit and seed production. Notably, its fruiting period usually occurs from July to October, during which precipitation is considered to have a significant effect on distribution predictions.

Originally native to the temperate zones of North America, *S. rostratum* thrives in climates similar to those found in the agro-pastoral ecotone of northern China, characterized by cool and moderately humid conditions. Therefore, its sensitivity to temperature and precipitation aligns with both its evolutionary adaptation and current invasive behavior in China. Among climatic factors, the annual mean temperature is one of the key environmental variables determining the geographic distribution of invasive species. Therefore, the trend of global warming may create favorable conditions for the survival and reproduction of *S. rostratum* [34]. This concurs with our findings, suggesting that continued global warming may promote the establishment and expansion of *S. rostratum* in areas previously deemed unsuitable. As climate change accelerates, regions with historically low suitability may become increasingly vulnerable to invasion, underlining the urgency of proactive monitoring and management strategies.

It is noteworthy that other environmental factors, such as topographic heterogeneity, soil properties, and geomorphological features, as well as land-cover and land-use patterns, may also play important roles in shaping the distribution and ecological impacts of invasive species. These factors not only affect habitat suitability but can also indirectly influence reproduction and spread by altering water availability, nutrient cycling, or microclimatic conditions. Given that this study primarily focuses on climate-driven variables, these factors were not systematically incorporated into the models, and, thus, the assessment of invasion potential remains somewhat limited.

### 3.3. Model Performance and Comparative Analysis

In this study, the Biomod2 ensemble modeling framework was employed to simulate the potential distribution of *S. rostratum* across China. The ensemble model achieved a TSS of 0.932 and an AUC value of 0.990, indicating excellent model performance. Compared to the best-performing single model (RF), the ensemble model yielded significantly higher accuracy and robustness, thereby offering a more reliable representation of the relationship between environmental factors and species occurrence. Species distribution model accuracy is highly sensitive to the sample size of occurrence data. Sampling biases—arising from methodology, timing, or location—can lead to over- or underrepresentation in certain regions, while human-mediated introductions may further distort observed distributions. Validation with field survey data is, therefore, essential. Future research should not only focus on improving the completeness and representativeness of species distribution data while accounting for anthropogenic factors, but also incorporate key environmental variables such as topography, soil, geomorphology, land cover, and land use into the modeling framework. Such integration would provide a more comprehensive understanding of the ecological drivers of species distribution. In our subsequent work, we plan to further refine the model to enhance the reliability and applicability of the prediction outcomes.

In recent years, ensemble modeling approaches such as Biomod2 have been widely adopted in predicting the potential distribution of invasive species. For example, Kou et al. [35] demonstrated that the ensemble modeling approach based on Biomod2 effectively predicted the suitable habitat distribution of *Lactuca serriola* L. and identified the key environmental variables driving its spread. This ensemble model significantly outperforms single models in terms of predictive accuracy and stability, thereby providing a more robust tool for the management and control of invasive plant species. Similarly, Tao et al. [36] developed an ensemble model to predict the suitable habitats of the invasive species *Pomacea canaliculata*. The results demonstrate that the ensemble model significantly outperformed individual models in predictive accuracy. In a previous study based on a single maximum entropy (MaxEnt)model, the potential suitable habitats of *S. rostratum* were primarily concentrated in northeast and northwest China [31]. Our findings, based on the Biomod2 ensemble framework, align closely with these earlier results, confirming that northeastern and northwestern China remain key distribution zones for this invasive species.

Furthermore, projections under future climate scenarios suggest a gradual southward expansion of *S. rostratum*’s suitable habitats, especially under high greenhouse gas emission pathways. These findings highlight the potential for *S. rostratum* to invade new regions under climate change, underscoring the urgent need for early detection systems and targeted management strategies to mitigate ecological risks.

## 4. Materials and Methods

### 4.1. Occurrence Data Collection

**Figure 7:**
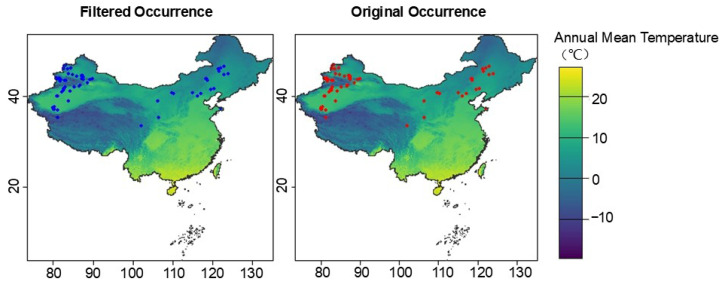
Occurrence records of *S. rostratum* used in the species distribution modeling. The left panel shows the original occurrence points (blue dots), while the right panel shows the spatially filtered occurrences (red dots) used for model calibration.

In this study, occurrence records of *S. rostratum* were obtained from the Chinese Virtual Herbarium (CVH, http://www.cvh.ac.cn/) (accessed on 29 October 2024) and the Global Biodiversity Information Facility (GBIF, https://www.gbif.org/) (accessed on 29 October 2024), as well as from the relevant literature published in the China National Knowledge Infrastructure (CNKI, https://www.cnki.net) (accessed on 29 October 2024) [37,38,39,40,41,42]. For records that provided specific localities but lacked geographic coordinates, latitude and longitude were determined using Baidu Maps (http://map.baidu.com) (accessed on 29 October 2024) and Google Earth to ensure spatial accuracy. The initial dataset comprised 119 occurrence records. To avoid spatial clustering and potential bias in model predictions, records with missing, erroneous, or ambiguous coordinates, as well as duplicates, were removed in R 4.2.1. The final dataset retained 85 unique, georeferenced occurrence points for subsequent modeling and spatial analyses (Figure 7). By integrating multi-source data and performing precise georeferencing, this study provides a solid foundation for constructing potential distribution models of *S. rostratum* while maximizing the representativeness and completeness of the occurrence dataset.

### 4.2. Environmental Variables Selection

A total of 55 environmental raster layers were initially selected from the WorldClim database, including monthly minimum temperature (°C), maximum temperature (°C), precipitation (mm), and 19 bioclimatic variables, all with a spatial resolution of 2.5 arc-minutes. Future climate data were sourced from the EC-Earth3-Veg model within the WorldClim database, based on different greenhouse gas concentration trajectories and socioeconomic development pathways. Specifically, four Shared Socioeconomic Pathways (SSPs) were used to represent a range of future climate conditions: SSP126 (low forcing), SSP245 (moderate forcing), SSP370 (moderate-to-high forcing), and SSP585 (high forcing). Environmental variables for China were extracted from the global datasets using ArcGIS 10.8. To reduce the risk of model overfitting caused by multicollinearity among predictor variables, we conducted a variance inflation factor (VIF) [43] analysis using the “raster” package in R 4.2.1. Only variables with VIF values less than 10 were retained for modeling. As a result, eight environmental variables were selected for the final model construction (Table 2).

### 4.3. Species Distribution Modeling

To predict the current and future potential distribution of *S. rostratum* in China, we employed the Biomod2 platform (version 3.5.1) in R 4.2.1—a comprehensive ensemble modeling framework for species distribution modeling, to predict the potential suitable habitats of *S. rostratum* under current conditions and for the 2030s (2020–2040), 2050s (2040–2060), 2070s (2060–2080), and 2090s (2080–2100). Biomod2 integrates multiple statistical and machine learning algorithms to quantify relationships between species occurrences and environmental variables, thereby predicting spatial distribution patterns [44,45]. Initially, we employed ten individual modeling algorithms embedded within the Biomod2 platform (Table 3). Due to the limited number of presence records, two sets of 500 pseudo-absence points (PA1 and PA2) were randomly generated to meet modeling requirements and better represent the potential absence of the species. During model calibration, 80% of occurrence records were randomly selected for training and the remaining 20% were reserved for testing. To reduce uncertainty and stochastic error, each algorithm was run twice on the same dataset, and model accuracy was evaluated using the TSS and the AUC. Only models with TSS > 0.8 were retained as candidate base models for ensemble modeling. Eligible models were then integrated using both EMca and exponential moving weighted mean (EMwmean) approaches in the Biomod2 package. The overall performance of the ensemble models was further assessed with TSS and ROC metrics. The final ensemble model, based on the optimal algorithmic combination, yielded high predictive accuracy and stability.

### 4.4. Data Processing and Visualization

Filtered occurrence records of *S. rostratum* and the selected climatic variables were incorporated into the ensemble model for prediction. To visualize invasion risk more intuitively, habitat suitability values were reclassified in ArcGIS 10.8 into four categories: unsuitable (0–0.2), low suitability (0.2–0.4), moderate suitability (0.4–0.6), and high suitability (0.6–1.0). The total suitable area was defined as the sum of low-, moderate-, and high-suitability classes, and suitability maps were generated accordingly. To identify potential expansion pathways, geographic centroids of suitable habitats were calculated for each future period, and centroid shift trajectories were mapped in ArcGIS 10.8. To further quantify spatial dynamics, binary change analysis was conducted in R, comparing current and future suitability to estimate changes in total and highly suitable areas, as well as expansion and contraction rates. In addition, variable importance analyses were performed, and heatmaps was generated in R to evaluate the relative contributions of climatic predictors.

## 5. Conclusions

This study employed the Biomod2 ensemble modeling framework to predict the potential distribution of *S. rostratum* in China under current and future climate conditions. The results show that suitable habitats are mainly concentrated in northeastern and northwestern China. Over time, the distribution centroid is projected to shift from Inner Mongolia toward southwestern Gansu Province, accompanied by a significant expansion of suitable areas, particularly into southern China. Temperature and precipitation were identified as the key climatic factors determining habitat suitability, and global warming is likely to further exacerbate its spread. Given the strong ecological adaptability and invasive potential of *S. rostratum*, it is urgent to strengthen monitoring in high-risk regions, establish climate-based early warning systems, and implement targeted control and ecological restoration strategies to mitigate its ecological impacts.

## Figures and Tables

**Table 1:** The changes in the suitable habitat range of *S. rostratum* under different climatic scenarios across different times periods.

Climate Scenario	Period	Total Suitable Area/km^2^	Highly Suitable Area/km^2^	Contraction Area/km^2^	Expansion Area/km^2^	Unchanged Area/km^2^	Contraction Rate/%	Expansion Rate/%
Current		1,191,586.55	600,215.76	——	——	——	——	——
SSP126	2030s	2,353,087.05	1,362,399.01	16,940	360,960	491,190	4.483	129.979
	2050s	2,419,584.54	1,468,682.39	77,740	774,410	143,940	9.123	16.891
	2070s	2,427,033.55	1,467,659.71	57,860	860,490	55,400	6.3	6.033
	2090s	2,530,792.36	1,636,708.54	28,480	887,410	132,720	3.11	14.491
SSP245	2030s	2,357,819.24	1,305,520.30	34,560	343,340	474,220	9.145	125.488
	2050s	2,544,239.44	1,646,379.74	41,150	776,410	250,480	5.033	30.638
	2070s	2,680,518.58	1,946,596.01	22,520	1,004,370	212,410	2.193	20.685
	2090s	2,620,111.73	1,884,886.90	117,840	1,098,940	80,030	9.685	6.577
SSP370	2030s	2,391,533.69	1,334,178.87	33,910	343,990	489,980	8.973	129.659
	2050s	2,643,836.62	1,690,488.06	28,470	805,500	250,350	3.414	30.019
	2070s	2,810,975.55	2,053,106.81	96,280	959,570	326,470	9.119	30.92
	2090s	2,939,625.48	2,035,003.91	133,550	1,152,490	124,550	10.385	9.685
SSP585	2030s	1,933,157.77	1,146,210.69	47,600	330,300	385,810	12.596	102.093
	2050s	2,680,658.82	1,921,309.23	26,540	689,570	51,060	3.706	71.314
	2070s	2,850,610.42	1,387,471.26	470,890	729,370	14,570	39.232	12.153
	2090s	2,976,804.38	1,822,833.93	64,320	810,920	330,250	7.349	37.733

**Table 2:** Environmental variables used in the model.

Type	Variable	Description	VIF
Bioclimatic variables	Bio_3	Isothermality (BIO2/BIO7 × 100)	2.474489
	Bio_9	Mean temperature of driest quarter	6.236408
	Bio_15	Precipitation seasonality (coefficient of variation)	4.626837
Precipitation	Prec_1	Precipitation in January	3.350388
	Prec_6	Precipitation in June	5.838437
	Prec_9	Precipitation in September	4.605355
Temperature	Tmax_7	Maximum temperature in July	2.787535
	Tmin_12	Minimum temperature in December	4.164368

**Table 3:** Species distribution model of Biomod2 platform.

Model Name	Model Code	References
Generalized linear model	GLM	Nelder et al. [46]
Gradient boosting machine	GBM	Friedman [47]
Generalize additive model	GAM	Hastie [48]
Multivariate adaptive regression spline model	MARS	Zakeri et al. [49]
Classification tree analysis model	CTA	Yarnold et al. [50]
Artificial neural networks model	ANN	Zupan [51]
Surface range envelop model	SRE	Kruithof et al. [52]
Flexible discriminant analysis model	FDA	Hastie et al. [53]
Random forest model	RF	Rigatti [54]
Maximum entropy model	MaxEnt	Phillips et al. [55]

## Data Availability

The authors do not have permission to share data. The data are not publicly available due to privacy and confidentiality concerns.
